# Branched chain amino acid transaminase 1 (*BCAT1*) is overexpressed and hypomethylated in patients with non-alcoholic fatty liver disease who experience adverse clinical events: A pilot study

**DOI:** 10.1371/journal.pone.0204308

**Published:** 2018-09-28

**Authors:** Kara Wegermann, Ricardo Henao, Anna Mae Diehl, Susan K. Murphy, Manal F. Abdelmalek, Cynthia A. Moylan

**Affiliations:** 1 Division of Gastroenterology, Department of Medicine, Duke University Health System, Durham, NC, United States of America; 2 Biostatistics and Bioinformatics, Duke University, Durham, NC, United States of America; 3 Department of Obstetrics and Gynecology, Duke University Health System, Durham, NC, United States of America; 4 Department of Medicine, Durham Veterans Affairs Health System, Durham, NC, United States of America; Medizinische Fakultat der RWTH Aachen, GERMANY

## Abstract

**Background and objectives:**

Although the burden of non-alcoholic fatty liver disease (NAFLD) continues to increase worldwide, genetic factors predicting progression to cirrhosis and decompensation in NAFLD remain poorly understood. We sought to determine whether gene expression profiling was associated with clinical decompensation and death in patients with NAFLD, and to assess whether altered DNA methylation contributes to these changes in gene expression.

**Methods:**

We performed a retrospective analysis of 86 patients in the Duke NAFLD Clinical Database and Biorepository with biopsy-proven NAFLD whose liver tissue was previously evaluated for gene expression and DNA methylation using array based technologies. We assessed the prospective development of liver and cardiovascular disease related outcomes, including hepatic decompensation as identified by the development of ascites, hepatic encephalopathy, hepatocellular carcinoma, or variceal bleeding as well as stroke and myocardial infarction via medical chart review.

**Results:**

Of the 86 patients, 47 had F0-F1 fibrosis and 39 had F3-F4 fibrosis at index liver biopsy. Gene expression probe sets (n = 54,675) were analyzed; 42 genes showed significant differential expression (p<0.05) and a two-fold change in expression between patients with and without any outcome. Two expression probes of the branched chain amino-acid transaminase 1 (*BCAT1*) gene were upregulated (p = 0.02; fold change 2.1 and 2.2 respectively) in patients with a clinical outcome. Methylation of three of the 34 *BCAT1* CpG methylation probes were significantly inversely correlated with *BCAT1* expression specific to the probes predictive of clinical deterioration.

**Conclusion:**

We found differential gene expression, correlated to changes in DNA methylation, at multiple *BCAT1* loci in patients with cardiovascular outcomes and/or hepatic decompensation. BCAT1 catalyzes the transformation of alpha-ketoglutarate to glutamate and has been linked to the presence and severity of NAFLD, possibly through derangements in the balance between glutamate and alpha-ketoglutarate. Given the potential for *BCAT1* to identify patients at risk for poor outcomes, and the potential therapeutic implications, these results should be validated in larger prospective studies.

## Introduction

The prevalence of non-alcoholic fatty liver disease (NAFLD) continues to increase worldwide resulting in significant morbidity and mortality [1]. NAFLD is currently the second most common reason for liver transplantation, but is projected to become the most common in the coming years [2]. However, the clinical course of individuals with NAFLD can vary dramatically; patients with simple steatosis may never experience liver dysfunction, while a subset develops inflammation and fibrosis, thereby increasing their risk for cirrhosis and complications of end-stage liver disease.

Currently, providers use a combination of clinical and laboratory data, imaging, fibrosis scoring systems and even histology to determine which NAFLD patients have advanced fibrosis and thus increased risk for poor outcomes. For example, obesity, advanced age, diabetes mellitus (DM), smoking and menopausal status have all been associated with advanced NAFLD fibrosis [3, 4]. Several scoring systems such as the NAFLD Fibrosis Score, FIB-4 and BARD score help clinicians estimate fibrosis stage [5–8]. Other algorithms such as Fibrosure and Fibrospect II and imaging modalities such as transient elastography and magnetic resonance elastography (MRE) can assist with investigating fibrosis, but these are expensive and not universally available [9]. Liver biopsy is considered the gold standard for fibrosis assessment, but is also expensive and invasive and carries the risk of complications. A major barrier exists in that while these data inform providers about fibrosis and cirrhosis, none of these predict fibrosis *progression*, the ultimate determinant of outcomes. Additionally, once cirrhosis is established, determining which patients will develop decompensation or other complications remains difficult. Identifying novel predictors for decompensation or important clinical outcomes such as cardiovascular disease could help clinicians target intensive management strategies for higher risk patients and potentially even inform future novel treatment targets.

Gene expression changes in liver and other tissues can differentiate NAFLD patients from those without NAFLD. These changes have largely been identified via candidate gene studies[10]. Previously, we conducted a cross sectional analysis revealing a 64-gene expression profile that accurately differentiated mild from severe NAFLD fibrosis histology independent of clinical factors [11]. Subsequently, we identified a group of genes and pathways whose expression in severe NAFLD was significantly inversely associated with DNA methylation [12]. Given the association of these genes with fibrosis and cirrhosis, we hypothesized that there would be be a group of genes also predictive of poor outcomes of NAFLD. The objectives of the present study were first to determine whether gene expression profiling at the time of liver biopsy is associated with the development of clinical decompensation and death, and second, to determine if DNA methylation changes were correlated with identified changes in gene expression. The eventual goal is to identify molecular pathways linked to NAFLD-related morbidity and mortality that could inform non-invasive markers able to predict poor outcomes in NAFLD, even prior to the development of advanced fibrosis or cirrhosis.

## Methods

### Patients and clinical data

We performed a retrospective analysis of patients with biopsy-proven NAFLD whose liver tissue was previously evaluated for gene expression and DNA methylation using array based technologies [11, 12]. Patients are part of the Duke University Health System (DUHS) NAFLD Biorepository, which contains frozen liver biopsy specimens, blood, and clinical data from patients undergoing diagnostic liver biopsy to grade and stage severity of NAFLD as part of standard of care. For study purposes, NAFLD was defined as: (1) presence of ≥ 5% hepatic steatosis on liver biopsy; (2) absence of histologic and serologic evidence for other chronic liver disease in a patient with risk factors for the metabolic syndrome. Patients were excluded from the biorepository if they were pregnant, unable to consent for liver biopsy, had a contraindication to liver biopsy, or had evidence of other etiologies of chronic liver disease (heavy alcohol use (>20 grams/day), detectable hepatitis C virus RNA level, or positive hepatitis B surface antigen). The biorepository is approved by the Duke Institutional Review Board, with patients consented for genomic analyses of specimens. Details of the histologic analysis and demographic information have been previously published [11]. Additional approval for clinical outcomes investigation was obtained for this study (Duke eIRB Pro00065463).

Baseline demographic information including height, weight, body mass index (BMI, kg/m^2^), age, gender, race, smoking status, comorbidities, and laboratory studies including fasting lipids, glucose, hemoglobin A1c (HbA1c), liver aminotransferases, and measures of liver synthetic function were collected on all patients within 3–6 months of liver biopsy. Methods for baseline data collection have been described previously [11]. Briefly, this was performed via patient questionnaires and manual data abstraction from the electronic medical record.

### Study period and outcomes

The follow up period for each patient was defined as the time of index liver biopsy until the occurrence of first clinical event, death, liver transplantation (LT), loss to follow up, or July 1, 2015, whichever came first. Outcomes were abstracted via manual review of the electronic medical record. Clinical deterioration/outcomes were defined as evidence of hepatic decompensation including the development of ascites (confirmed on imaging) hepatic encephalopathy (requiring treatment with lactulose and/or rifaximin), diagnosis of hepatocellular carcinoma, or variceal bleeding. Stroke and myocardial infarction were assessed using discharge summaries and problem lists. Patients were excluded if they had evidence of clinical decompensation at the time of liver biopsy or had previously undergone LT.

### Gene expression analysis

Details of the hepatic gene expression analysis have been described previously [11]. Briefly, liver biopsy samples were snap frozen in liquid nitrogen and stored at −80°C. RNA was isolated from using the AllPrep Micro Kit (QIAGEN, Valencia, CA) according to the manufacturer’s instructions after samples were thawed in *RNAlater-*ICE at -20°C (Applied Biosystems/Ambion, Austin, TX). Microarray hybridization was performed on Affymetrix Human Genome U133 Plus 2.0 GeneChip arrays (Affymetrix, Santa Clara, CA), using MessageAmp Premier (Applied Biosystems, Foster City, CA) for RNA amplification and hybridization. A total of 54,675 probes were considered for analyses after standard RMA normalization.

### DNA methylation analysis

Details regarding the DNA methylation analysis, quality control, and normalization procedures on frozen liver biopsy samples from the same patients have been described [12]. Briefly, DNA was bisulfite modified using the Zymo EZ DNA Methylation Kit. The bisulfite modified genomic DNAs were submitted to Expression Analysis (Research Triangle Park, NC) and assayed on the Illumina HumanMethylation450 beadchip data. From a total of 485,512 CpG sites, 34 CpG sites associated with BCAT1 were considered for analyses after quantile normalization using the minfi R package [13].

### Statistical analysis

Generalized linear models were used to quantify the association between gene expression and the composite outcome, clinical variables and fibrosis stage. The link function of the generalized linear model was set according to the nature of the independent variable (continuous, binary or ordinal). The primary model was controlled for age, BMI, DM, fibrosis stage, and batch, with the composite outcome of death, liver-related morbidity or cardiovascular disease as the dependent variable of interest and gene expression the independent variable. *P*-values < 0.05 with a two-fold or greater change in gene expression were considered statistically significant, unless otherwise specified. Correlations between gene expression probes and methylation CpG sites were estimated via Spearman’s rank-order correlation. All statistical analyses were performed in Matlab (MathWorks, Inc).

## Results

### Patient characteristics

Eighty-six patients with high quality hepatic gene expression data were included in the study. Patients were divided by fibrosis stage as part of the original gene expression analysis; 47 patients had fibrosis stage F0 or F1 and 39 had fibrosis stage F3 or F4 (**Table 1**). Most patients were female (67%, n = 58), white (89%, n = 77), and obese with a median BMI of 36 kg/m^2^. BMI did not differ by fibrosis stage. Median age at study entry was 53 years (range, 27 to 81 years). Patients with more advanced stages of fibrosis were significantly older than those with milder stages of fibrosis (p = 0.04). Thirty-six (41.9%) patients had a diagnosis of DM and this was significantly more common in those with advanced stages of fibrosis (p = 0.001).

**Table 1:** Patient characteristics at baseline. P-values reflect fibrosis stage comparisons.

	Overall (n = 86)	Mild NAFLD (n = 47)		Advanced NAFLD (n = 39)		p-value
Fibrosis Stage		F0 (n = 17)	F1 (n = 30)	F3 (n = 30)	F4 (n = 9)	
**Gender (% female)**	58 (67.4)	11 (64.7)	18 (60.0)	2 (73.3)	7 (77.8)	0.63
**Age, mean**±**SD**	50.9 ± 10.6	51.1 ± 9.1	50.6 ± 10.7	48.3 ± 10.8	59.7 ± 8.7	0.50
**Race**						0.64
**White (%)**	77 (89.5)	15 (88.2)	27 (90.0)	27 (90.0)	8 (88.9)	
**Black (%)**	6 (7.0)	2 (11.8)	1 (3.3)	2 (6.7)	1 (11.1)	
**Body Mass Index (kg/m2) mean** ± **SD**	36.3 ± 8.9	35.4 ± 8.3	35.8 ± 8.9	36.9 ± 7.9	37.5 ± 13.2	0.43
**Diabetes Mellitus (%)**	36 (41.9)	4 (23.5)	7 (23.3)	18 (60.0)	7 (77.8)	**0.001**
**Hyperlipidemia (%)**	55 (63.9)	14 (82.4)	19 (63.3)	18 (60.0)	4 (44.4)	0.24
**Hypertension (%)**	54 (62.8)	11 (64.7)	16 (53.3)	19 (63.3)	8 (88.9)	0.28
**Smoking (%)**	6 (7.7)	2 (11.8)	1 (3.8)	3 (11.5)	0	0.52
**NAFLD Activity Score (NAS) > = 5**	34 (39.5)	2 (11.8)	11 (36.7)	19 (63.3)	2 (22.2)	**0.003**
**Hemoglobin A1c (%), mean ± SD**	6.32 ± 1.0	5.9 ± 0.5	6.1 ± 1.2	6.6 ± 1.2	6.5 ± 0.8	**0.033**

SD, standard deviation

### Outcomes

Follow up from time of liver biopsy to outcome or study end ranged from 0 to 8.2 years (2,997 days) and seventy-five patients (87%) had follow up for at least one year. Over a median follow up of 5.4 years (1,986 days), four patients experienced five clinical outcomes; one had esophageal variceal bleeding and a stroke, two others experienced strokes and one developed hepatic decompensation alone in the form of hepatic encephalopathy. All four patients with outcomes were white (non-Hispanic) women and 3 of 4 had advanced fibrosis stage at study entry. One patient previously had breast cancer, while the other three had family histories of lung cancer (two in first degree relatives, one in a second degree relative). Characteristics of subjects with outcomes are shown in **Table 2**. All four patients with any outcome were analyzed together for the gene expression and DNA methylation analyses.

**Table 2:** Characteristics of patients who experienced liver or cardiovascular related events during follow up.

Subject	Race and Gender	Age at Study Entry / Liver Biopsy	Fibrosis Stage at Study Entry	Comorbidities	Type of Outcome	Time to Outcome
1	White Female	40	3	Current Smoker	Hepatic encephalopathy	5.6 years
2	White Female	71	4	Diabetes Hypertension	Variceal bleed Stroke	3.5 years
3	White Female	57	1	Diabetes Hypertension Current smoker	Stroke	5.0 years
4	White Female	52	4	Diabetes Hypertension Current smoker	Stroke	6.4 years

### Gene expression

**Fig 1:**
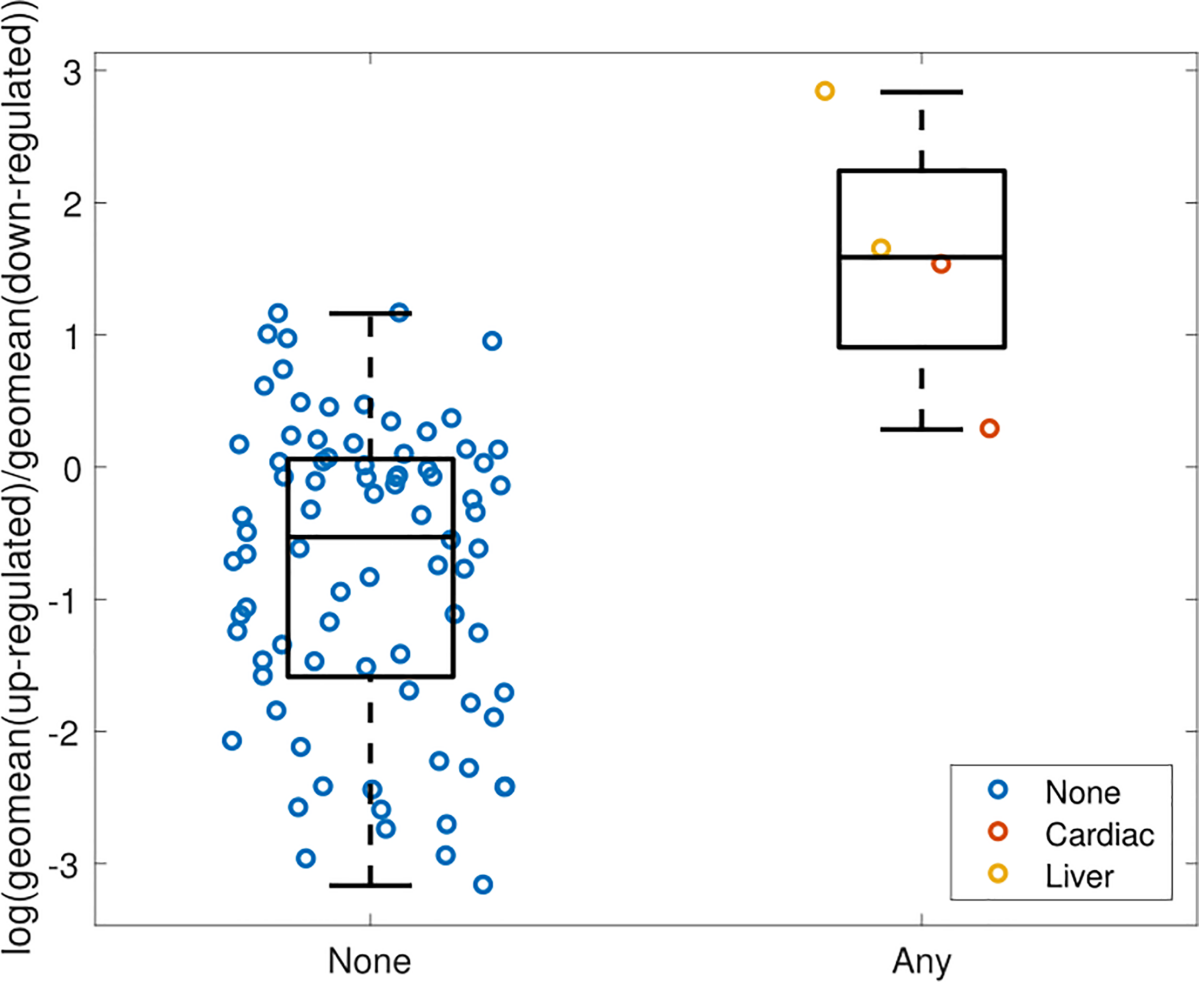
The 42 gene signature discriminates between NAFLD patients with and without clinical outcomes. The signature value (y-axis) is calculated as the geometric mean of upregulated probesets minus the geometric mean of downregulated probesets in log domain. Subjects (circles) are grouped by outcome (x-axis).

**Fig 2:**
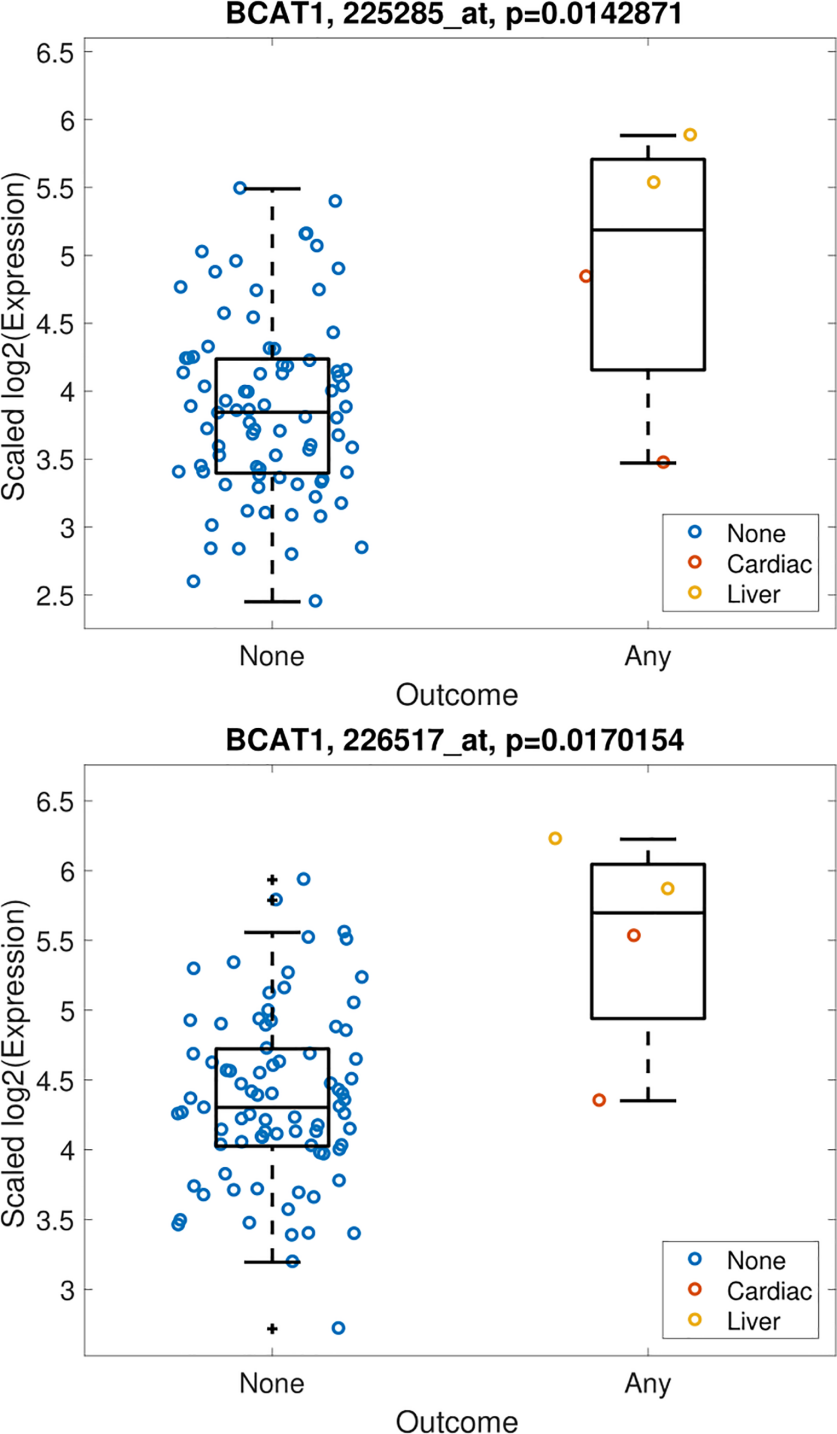
Baseline *BCAT1* expression is upregulated in NAFLD patients with clinical outcomes. Scaled expression values in log domain (y-axis) and subjects (circles) grouped by outcome (x-axis).

To evaluate the association between hepatic gene expression and clinical deterioration, we used a composite measure of hepatic decompensation, death and cardiovascular events due to the limited number of outcomes during follow up. Of 54,675 gene expression probe sets analyzed, 42 genes showed significant differential expression (*p*<0.05) and a two-fold change in expression between patients with and without any outcome. Of these, 35 probe sets were upregulated and 7 were downregulated. The combined 42 gene signature was able to discriminate between patients with and without outcomes **(Fig 1, S1 Fig—patients shown by fibrosis stage)**. Five genes/probes were significantly differentially expressed with *p* < 0.02 and a two-fold change in expression in patients with and without an outcome. 1,847 were significantly differentially expressed at the *p* < 0.05 level, regardless of fold-change. Two probes (226517_at, 225285_at) of the branched chain amino-acid transaminase 1 (*BCAT1*) gene were upregulated (*p*<0.02; fold change 2.1 and 2.2 respectively) in patients with a clinical outcome. Other differentialy expressed genes of interest include those involved in apoptosis signaling (*BCL2A1*), one-carbon metabolism (*MTHFD2*), and cell adhesion (*ITGAM*, *CLDN11*). A complete list of the significantly differentially expressed genes appears in **Table 3**. As shown in **Fig 2**, *BCAT1* expression was more highly upregulated in patients with liver related morbidity as compared to patients with a cardiovascular event or without any outcome.

**Table 3:** Differentially expressed genes associated with clinical outcomes. All probes have a false discovery rate of 0.65.

ProbeSet	Gene_Symbol	Gene Name	Dir	FC	PV
225285_at	BCAT1	branched chain amino-acid transaminase 1, cytosolic	↑	2.13	0.01
226517_at	BCAT1	branched chain amino-acid transaminase 1, cytosolic	↑	2.20	0.02
205786_s_at	ITGAM	integrin, alpha M (complement component 3 receptor 3 subunit)	↑	2.33	0.02
206134_at	ADAMDEC1	ADAM-like, decysin 1	↑	3.47	0.02
204419_x_at	HBG1 /// HBG2	hemoglobin, gamma A /// hemoglobin, gamma G	↑	2.03	0.02
207815_at	PF4V1	platelet factor 4 variant 1	↑	3.08	0.02
205681_at	BCL2A1	BCL2-related protein A1	↑	2.24	0.02
203196_at	ABCC4	ATP-binding cassette, sub-family C (CFTR/MRP), member 4	↑	2.09	0.02
228335_at	CLDN11	claudin 11	↑	2.65	0.02
201601_x_at	IFITM1 /// IFITM2	interferon induced transmembrane protein 1 /// interferon induced transmembrane protein 2	↑	2.03	0.03
211663_x_at	PTGDS	prostaglandin D2 synthase 21kDa (brain)	↑	2.07	0.03
1554018_at	GPNMB	glycoprotein (transmembrane) nmb	↑	2.54	0.03
34210_at	CD52	CD52 molecule	↑	2.61	0.03
211538_s_at	HSPA2	heat shock 70kDa protein 2	↑	2.32	0.03
203088_at	FBLN5	fibulin 5	↑	2.04	0.03
205624_at	CPA3	carboxypeptidase A3 (mast cell)	↑	2.83	0.03
225681_at	CTHRC1	collagen triple helix repeat containing 1	↑	2.44	0.03
212915_at	PDZRN3	PDZ domain containing ring finger 3	↑	2.10	0.03
204963_at	SSPN	sarcospan	↑	2.47	0.03
214837_at	ALB	albumin	↓	0.48	0.04
208651_x_at	CD24	CD24 molecule	↑	3.09	0.04
244181_at	—	—	↓	0.48	0.04
204661_at	CD52	CD52 molecule	↑	2.22	0.04
201761_at	MTHFD2	methylenetetrahydrofolate dehydrogenase (NADP+ dependent) 2, methenyltetrahydrofolate cyclohydrolase	↑	2.13	0.04
1568920_at	—	—	↓	0.49	0.04
213975_s_at	LYZ	lysozyme	↑	2.18	0.04
204361_s_at	SKAP2	src kinase associated phosphoprotein 2	↑	2.05	0.04
206456_at	GABRA5	gamma-aminobutyric acid (GABA) A receptor, alpha 5	↑	2.51	0.04
242169_at	BHMT2	betaine—homocysteine S-methyltransferase 2	↓	0.47	0.04
214829_at	AASS	aminoadipate-semialdehyde synthase	↓	0.42	0.04
207332_s_at	TFRC	transferrin receptor	↑	2.89	0.04
243929_at	—	—	↓	0.49	0.04
223235_s_at	SMOC2	SPARC related modular calcium binding 2	↑	2.60	0.04
214693_x_at	NBPF10 /// NBPF14 /// NBPF26 /// NBPF9	neuroblastoma breakpoint family, member 10 /// neuroblastoma breakpoint family, member 14 /// neuroblastoma breakpoint family, member 26 /// neuroblastoma breakpoint family, member 9	↓	0.49	0.04
230422_at	FPR3	formyl peptide receptor 3	↑	2.10	0.04
214768_x_at	IGKC /// IGKV2-28 /// IGKV2-28 /// IGKV2D-28 /// IGKV2D-28	immunoglobulin kappa constant /// immunoglobulin kappa variable 2–28 /// — /// immunoglobulin kappa variable 2D-28 /// —	↑	2.29	0.04
202949_s_at	FHL2	four and a half LIM domains 2	↑	2.19	0.05
212187_x_at	PTGDS	prostaglandin D2 synthase 21kDa (brain)	↑	2.27	0.05
201744_s_at	LUM	lumican	↑	2.68	0.05
225105_at	C12orf75	chromosome 12 open reading frame 75	↑	2.90	0.05
204774_at	EVI2A	ecotropic viral integration site 2A	↑	2.07	0.05
223484_at	C15orf48	chromosome 15 open reading frame 48	↑	2.67	0.05

Dir, Direction of gene expression change associated with clinical outcome; PV, uncorrected p-value

Next, we evaluated the correlation between *BCAT1* expression and other markers of advanced liver disease. After controlling for age, BMI, fibrosis stage, and DM, the two *BCAT1* gene probes (226517_at, 225285_at) were significantly upregulated with several histologic markers of advanced disease, including higher fibrosis stage, higher NAFLD activity scores, steatosis, lobular inflammation and ballooning. *BCAT1* expression was also significantly upregulated with clinical factors such as higher glycosylated hemoglobin (HbA1c), alanine aminotransferase (ALT) and aspartate aminotransferase (AST) values (**S1 Table**) but not BMI, total cholesterol, total bilirubin, albumin, creatinine or a diagnosis of hypertension (results not shown).

### DNA methylation and *BCAT1*

Previously, we established that patterns in human hepatic DNA methylation can distinquish patients with advanced NAFLD fibrosis from those with mild NAFLD fibrosis [12]. Moreover, we found that for many genes, DNA methylation was significantly inversely correlated with gene expression and thus possibly involved in regulation of that expression. Given the association of hepatic gene expression of *BCAT1* and NAFLD outcomes, we investigated whether DNA methylation is specifically associated with altered gene expression of *BCAT1* and advanced fibrosis and/or NAFLD related morbidity and mortality as a way of validating our gene expression results.

**Fig 3:**
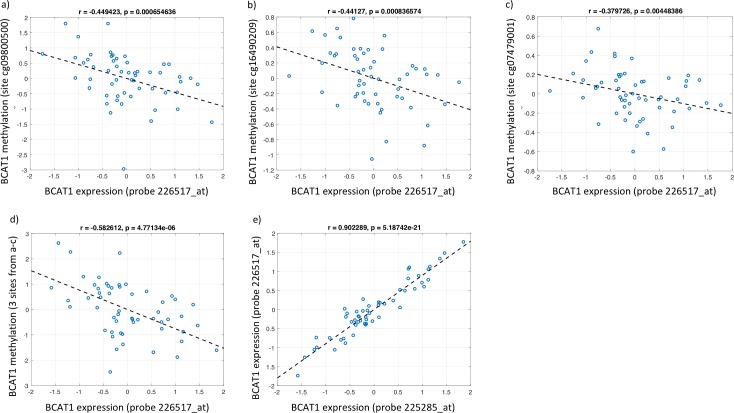
Correlation between BCAT1 gene expression and methylation. **3a)– 3c)** show the significant inverse correlation between gene expression for one probe on *BCAT1* (226517_at) and methylation at three separate CpG sites on *BCAT1* (cg09800500, cg16490209 and cg07479001). Negative correlation is highlighted by the linear fit (dashed line) with corresponding correlation coefficients (r values) and statistical significance. **3d)** Correlation between gene expression at the previously mentioned *BCAT1* probe (226517_at) and DNA methylation improves when the three signficant CpG sites are combined (summed) for analysis. **3e)** Two gene expression probes on *BCAT1* (226517_at and 225285_at) are well correlated with one another. For all figures, normalized gene expression and methylation M values are shown. For 3a)- 3c), each point represents a paired gene expression and methylation sample. M values are inversely correlated with gene expression values as expected.

Epigenome-wide DNA methylation data was available for a subgroup of patients with gene expression and outcome data (n = 55; 3 outcomes). Methylation of three of the 34 CpG sites on the Illumina 450K beadchip annotated as *BCAT1* (cg09800500, cg07479001, cg1649029) was significantly inversely correlated with the two *BCAT1* expression probes predictive of clinical deterioration (**Table 4, Fig 3**). Given the small sample size, these three CpG sites were not significantly associated with the composite outcome, but were inversely correlated (*i*.*e*, the sites are hypomethylated in subjects with outcomes). Utilizing the three significantly correlated CpG sites, we assessed the association between methylation and histologic and clinical factors of advanced NAFLD as well as correlation between methylation and gene expression. *BCAT1* hypomethylation was significantly inversely associated with higher fibrosis stage, more hepatocyte ballooning and more lobular inflammation (**S1 Table**).

**Table 4:** Correlation matrix of BCAT1 gene expression and DNA methylation at Illumina CpG sites with *p*-value shown in parentheses.

	cg09800500	cg16490209	cg07479001
226517_at	-0.45 (5.8 x 10–4)	-0.44 (7.5 x 10–4)	-0.38 (4.2 x 10–3)
225285_at	-0.38 (4.2 x 10–3)	-0.45 (6.5 x 10–4)	-0.34 (1.1 x 10–2)

## Discussion

Given an aging population and its association with obesity and diabetes, the worldwide burden of NAFLD continues to rise. Models forcast continued increased prevalence of not only NAFLD but NASH, cirrhosis and decompensation through 2030 [14]. These unfortunate statistics leave providers grappling with who and how to effectively screen and monitor patients for complications. While it is well known that NASH fibrosis stage correlates with long-term liver related events and death, accurate markers able to predict such events in individuals are lacking. This unmet need can result in poor utilization of resources, missed opportunities for intervention and counseling, and inability to potentially prevent complications such as cardiovascular disease or liver-related decompensation and death.

As a first step towards developing markers to predict NAFLD outcomes, we sought to determine whether specific alterations in hepatic gene expression and methylation at time of index biopsy could identify patients who later experience decompensation or cardiovascular events. We identified 42 differentially expressed genes in the liver at the time of liver biopsy in patients with a future event. The 42 gene signature effectively discriminated between those patients with an outcome (particularly liver decompensation) versus those without. Interestingly, the top two differentially expressed probes in the signature belong to the gene, *BCAT1*. In this study, *BCAT1* was upregulated and hypomethylated in NAFLD patients with clinical decompensation or cardiovascular events compared to those without.

The unbiased finding of *BCAT1*’s association with clinical deterioration in NAFLD is interesting and relevant. BCAT1 is a cytosolic enzyme that initiates the breakdown of essential branched chain amino acids (BCAA) (leucine, isoleucine and valine) by catalyzing the transformation of α-ketoglutarate to glutamate resulting in the respective branched chain α-keto acids (BCKAs). Thus, BCAT1 along with the other BCAT enzymes are important regulators of metabolism through their influence on the tricarbocylic acid (TCA) cycle and oxidative phosphorylation [15]. *BCAT1* expression has been reported in several tissues including embryonic tissues, brain, ovary, kidney and to a lesser extent in liver, intestine and pancreas [16].

The role of *BCAT1* and BCAAs in NASH has been investigated previously. In a small cohort of patients with biopsy proven NAFLD, increased hepatic *BCAT1* expression correlated with percentage of steatosis [17]. More recently, elevated levels of hepatocellular cytosolic *BCAT1* mRNA and protein were found in NASH patients, whereas those with simple steatosis had no BCAT1 expression [18]. It is unclear, however, whether extrahepatic enzymatic sources may be contributing, or if this abnormal BCAT activity is isolated to NASH livers [19, 20]. In addition, a recent large study assessing the relationship between serum metabolites and future development of NAFLD found that branched chain amino acids were highly associated with NAFLD development, suggesting that discovery of a serum biomarker related to this pathway may be feasible [21].

The mechanisms by which BCAT1 influences outcomes in NAFLD are not precisely understood, but there are several compelling hypotheses. BCAAs mediate signaling in liver tissue, and patients with advanced chronic liver disease have low BCAA levels in liver tissue [22]. In animal models, BCAA supplementation improves steatosis [23]. In humans, BCAA supplementation may improve insulin resistance in patients with NASH cirrhosis [24]. In one study using genomic algorithms to discover NASH biomarkers, serine deficiency and excess glutamate were highly significant. BCAT1 was the only upregulated enzyme linked to glutamate [25]. Potentially, simultaneous upregulation of BCAT1 and downregulation of phosphoserine phosphatase (PSPH) is indicative of an intracellular imbalance of α-ketoglutarate and glutamate, leading to accumulation of glutamate. This points to *BCAT1* as a therapeutic target to address metabolic deranagements of NASH [25].

Beyond NASH, *BCAT1* appears to have oncogenic properties. *BCAT1* is upregulated and functionally required for several malignancies including glioblastoma, hepatocellular carcinoma (HCC), colorectal and breast cancers, and chronic myelogenous leukemia [26]. The metabolic role of *BCAT1* appears to be related to tissue of origin. In HCC, *BCAT1* expression was higher in tumor tissue compared to non-tumor tissue, and was associated with resistance to chemotherapy and poor prognosis [27–29]. Augmented amino acid metabolism is associated with clinical aggression in malignant gliomas and appears to sustain growth in breast cancer [30, 31]. Similar to our results, *BCAT1* is hypomethylated in some patients with colorectal cancer, and is being investigated as a peripheral biomarker [32].

The strengths of our study includes liver-biopsy proven NAFLD patients with long-term outcome data, an unbiased analysis of gene expression, and the use of DNA methylation data to corroborate the gene expression association we found with *BCAT1*. Our study also has several limitations. First, given the relatively short follow up and high percentage of patients with mild stages of liver fibrosis, we indentified only a small number of outcomes. This limited our statistical power. Second, we performed a retrospective analysis of clinical outcomes, and therefore could have missed diagnoses that occurred outside our center or to patients who were lost to followup. In order to further validate our findings, longer and larger prospective studies of NAFLD patients will need to be performed. Similarly, assessing non-invasive markers from blood or from circulating cells through metabolomics, microRNA or DNA in blood would be the next step in developing easy to obtain prognostic indicators. Whether *BCAT1* is upregulated in both liver tissue and peripheral blood is not clear, as it has been more frequently evaluated in liver. Finally, inclusion of metabolomics or genomic markers along with key clinical factors could allow development of an accurate prognostic risk index able to predict morbidity and mortality in NAFLD.

In conclusion, this pilot study suggests that perturbations in hepatic metabolism are associated with future poor outcomes in NAFLD patients. BCAA metabolism via *BCAT1* alterations are particularly important and may differentiate those patients at increased risk. Our study provides a nice first step in developing prognostic indicators of key clinical outcomes in NAFLD patients. The hope is that with continued investigation, these markers will allow for appropriate risk stratification, intervention and prevention of poor outcomes of NAFLD.

## Supporting information

S1 Table BCAT1 probe expression and CpG methylation are significantly correlated with several markers of more advanced NAFLD and metabolic syndrome. Regression coefficients (Coeff) quantify the association between the probe or CpG site and clinical characteristic. The sign of the coefficient indicates direction of the association.
(DOCX)

S1 Fig The 42 gene signature discriminates between NAFLD patients with and without clinical outcomes, and has similar discriminatory ability when subjects are stratified by fibrosis stage. The signature value (y-axis) is calculated as the geometric mean of upregulated probesets minus the geometric mean of downregulated probesets in log domain. Subjects are grouped by outcome (x-axis) and separated by fibrosis stage at baseline liver biopsy (circle: Stage F0-F1 Fibrosis, Mild; x: Stage F3-F4 Fibrosis, Advanced). Gene expression is up regulated in NAFLD patients with cardiovascular and liver-related outcomes.
(TIFF)

## Abbreviations

NAFLD: non-alcoholic fatty liver disease
BCAT1: branched chain amino-acid transaminase 1
DM: diabetes mellitus
MRE: magnetic resonance elastography
LT: liver transplantation
BMI: body mass index
ALT: alanine aminotransferase
AST: aspartate aminotransferase
BCAA: branched chain amino acids
BCKA: branched chain α-keto acids
