# Correction: Branched chain amino acid transaminase 1 (*BCAT1*) is overexpressed and hypomethylated in patients with non-alcoholic fatty liver disease who experience adverse clinical events: A pilot study

**DOI:** 10.1371/journal.pone.0212144

**Published:** 2019-02-05

**Authors:** The PLOS ONE Staff

There is information missing from the Data Availability section. The publisher apologizes for this error.

The complete, correct Data Availability section is: Gene expression and methylation data are available through GEO at GSE31803. Clinical data are available to researchers who meet the criteria for access to confidential data through the Duke NAFLD Biorepository (ying.wang@duke.edu, Data Manager) or the Corresponding Author (cynthia.moylan@duke.edu).
