# Supplementary material for: Branched chain amino acid transaminase 1 (BCAT1) is overexpressed and hypomethylated in patients with non-alcoholic fatty liver disease who experience adverse clinical events: A pilot study
Source: PLoS One. 2018 Sep 28;13(9):e0204308. doi: 10.1371/journal.pone.0204308 (PMC6161885; doi:10.1371/journal.pone.0204308)
Supplement: S1 Table — Regression coefficients (Coeff) quantify the association between the probe or CpG site and clinical characteristic. The sign of the coefficient indicates direction of the association. (DOCX) [file pone.0204308.s001.docx]

**S1 Table.** BCAT1 probe expression and CpG methylation are significantly correlated with several markers of more advanced NAFLD and metabolic syndrome. Regression coefficients (Coeff) quantify the association between the probe or CpG site and clinical characteristic. The sign of the coefficient indicates direction of the association.

|  | **BCAT1 gene probe IDs** | | | | **BCAT1 Illumina CpG site IDs** | | | | | |
| --- | --- | --- | --- | --- | --- | --- | --- | --- | --- | --- |
| Clinical Characteristic | **226517_at** | | **225285_at** | | **Cg09800500** | | **Cg16490209** | | **Cg07479001** | |
|  | Coeff | p-value | Coeff | p-value | Coeff | p-value | Coeff | p-value | Coeff | p-value |
| **Hemoglobin A1C** | 0.37 | **8x10-3** | 0.36 | **7x10-3** | -0.02 | 0.84 | -0.36 | 0.14 | -0.33 | 0.36 |
| **Alanine Aminotransferase (ALT)** | 36.93 | **1x10-4** | 32.27 | **5x10-4** | -15.41 | 0.19 | -54.57 | **0.036** | -49.61 | 0.20 |
| **Aspartate Aminotransferase (AST)** | 25.77 | **1x10-5** | 22.24 | **1x10-4** | -11.64 | 0.11 | -36.62 | **0.024** | -38.63 | 0.11 |
| **Thyroid Stimulating Hormone** | -0.42 | 0.078 | -0.46 | **0.040** | 0.16 | 0.38 | -0.03 | 0.93 | -0.06 | 0.91 |
| **Fibrosis (Early vs. Advanced)** | 1.79 | **5x10-4** | 1.41 | **1x10-3** | -2.29 | **1x10-3** | -1.96 | **0.033** | -3.78 | **0.016** |
| **Fibrosis stage** | 0.96 | **1x10-6** | 0.82 | **4x10-5** | -0.92 | **4x10-5** | -1.09 | **0.036** | -1.51 | 0.052 |
| **NASH is this NAFLD activity score>=5?** | 0.96 | **1x10-4** | 0.85 | **5x10-4** | -0.53 | 0.064 | -1.19 | **2x10-3** | -2.73 | **3x10-3** |
| **Steatosis** | 0.36 | **5x10-3** | 0.31 | **0.012** | -0.07 | 0.59 | -0.52 | 0.10 | -0.60 | 0.21 |
| **Lobular Inflammation** | 0.24 | **0.028** | 0.22 | **0.033** | -0.17 | 0.12 | -0.69 | **6x10-3** | -0.92 | **0.013** |
| **Ballooning** | 0.35 | **1x10-3** | 0.30 | **3x10-3** | -0.24 | 0.057 | -0.70 | **5x10-3** | -1.02 | **6x10-3** |
| **Any outcome** | 3.39 | **0.019** | 2.47 | **0.025** | -0.65 | 0.49 | -0.76 | 0.65 | -2.24 | 0.38 |
