# Supplementary figures and images for: Branched chain amino acid transaminase 1 (BCAT1) is overexpressed and hypomethylated in patients with non-alcoholic fatty liver disease who experience adverse clinical events: A pilot study
Source: PLoS One. 2018 Sep 28;13(9):e0204308. doi: 10.1371/journal.pone.0204308 (PMC6161885; doi:10.1371/journal.pone.0204308)

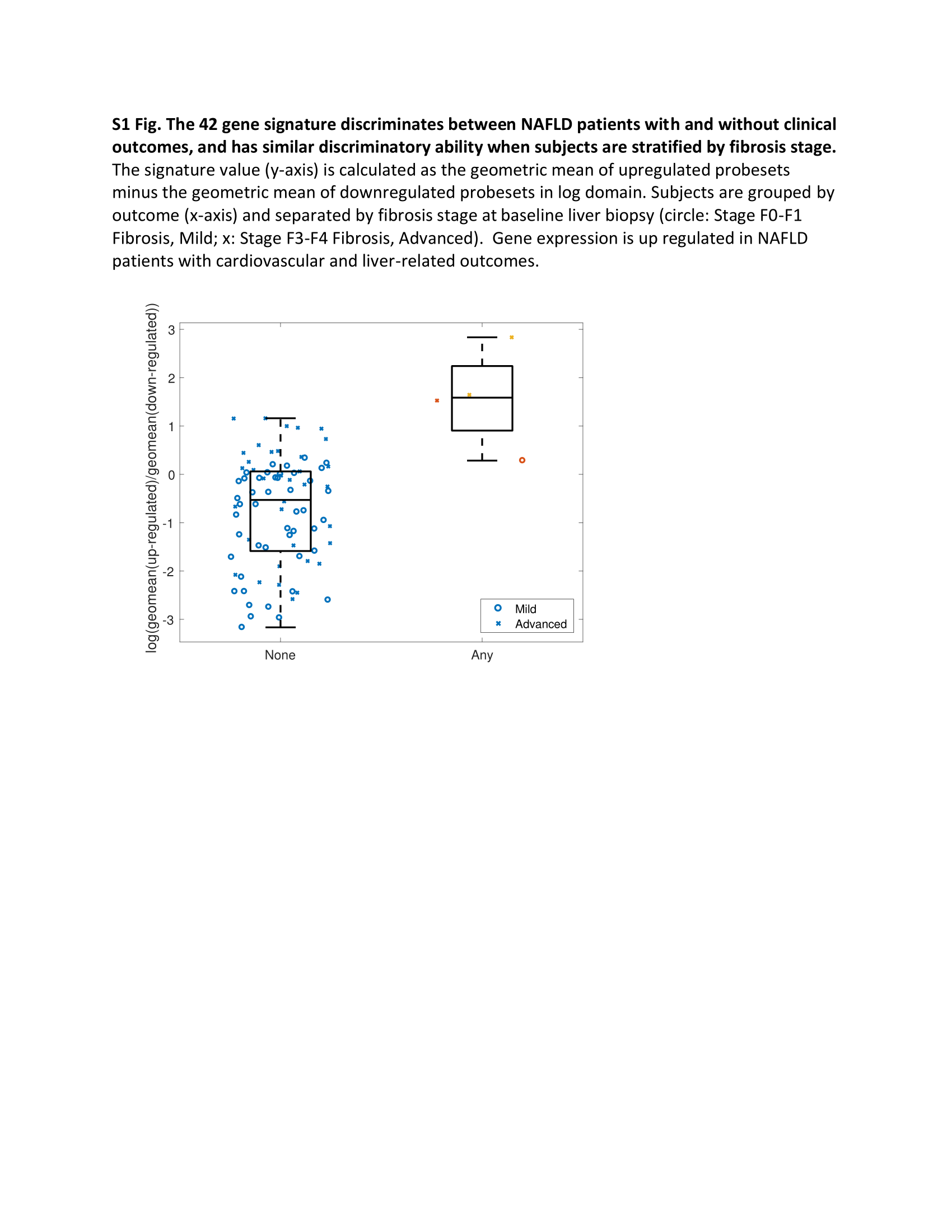

Supplement: S1 Fig — The signature value (y-axis) is calculated as the geometric mean of upregulated probesets minus the geometric mean of downregulated probesets in log domain. Subjects are grouped by outcome (x-axis) and separated by fibrosis stage at baseline liver biopsy (circle: Stage F0-F1 Fibrosis, Mild; x: Stage F3-F4 Fibrosis, Advanced). Gene expression is up regulated in NAFLD patients with cardiovascular and liver-related outcomes. (TIFF) [file pone.0204308.s002.tiff]
